# Improvement in Predicting the Post-Cracking Tensile Behavior of Ultra-High Performance Cementitious Composites Based on Fiber Orientation Distribution

**DOI:** 10.3390/ma9100829

**Published:** 2016-10-13

**Authors:** Myoung Sung Choi, Su-Tae Kang, Bang Yeon Lee, Kyeong-Taek Koh, Gum-Sung Ryu

**Affiliations:** 1 Department of Safety Engineering, Dongguk University-Gyeongju, 123 Dongdae-ro, Gyeongju, Gyeongbuk 38066, Korea; mschoi@dongguk.ac.kr; 2 Department of Civil Engineering, Daegu University, 201 Daegudae-ro, Jillyang, Gyeongsan, Gyeongbuk 38453, Korea; 3 School of Architecture, Chonnam National University, 77 Yongbong-ro, Buk-gu, Gwangju 61186, Korea; bylee@jnu.ac.kr; 4 Structural Engineering Research Institute, Korea Institute of Civil Engineering and Building Technology, 283 Goyangdae-Ro, Ilsanseo-Gu, Goyang, Gyeonggi 10223, Korea; ktgo@kict.re.kr (K.-T.K.); ryu0505@kict.re.kr (G.-S.R.)

**Keywords:** post-cracking behavior, fiber reinforcement, UHPCC, fiber orientation, bridging

## Abstract

In this paper, the post-cracking tensile behavior of Ultra-High Performance Cementitious Composites (UHPCC) was studied and an improved analytical model to predict the behavior depending on the fiber orientation distribution was proposed. Two different casting methods were adopted to estimate the influence of the casting method on the tensile behavior. The direct tensile test results showed that the post-cracking tensile behavior was considerably dependent on the casting method. The influence of the casting method was quantified by image analysis of the fiber distribution. The fiber orientation distribution obtained by image analysis may sometimes include considerable error according to the image resolution, which may cause inaccuracy when predicting the post-cracking tensile behavior based on the fiber orientation distribution. To overcome this dependency, the tensile bridging behavior by the fibers in UHPCC was simulated considering the obtained fiber orientation distribution as well as the number of fibers detected. The post-cracking behavior was then simulated by combining the bridging behavior and tension softening behavior of the matrix. The approach adopted in this study to simulate the post-cracking behavior of UHPCC showed good agreement with the experimental results.

## 1. Introduction

Ultra-High Performance Cementitious Composites (UHPCC), which is now one of several major topics related to recent concrete technology, exhibit very high compressive strength over 200 MPa as well as excellent durability [1,2,3,4,5]. With the help of short steel fibers, the inherent brittleness of high strength concrete can be overcome and sufficient ductility can be obtained in both compression and tension. This results in a high tensile strength of more than 10 MPa in direct tension and 35 MPa or more in bending [6,7,8,9]. Owing to its superior mechanical properties and durability, UHPCC is applied increasingly to a range of structures including bridges, buildings, etc.

The most beneficial effect of incorporating short fibers into a cement matrix is the improvement in tensile strength and ductility, even though this is significantly dependent on the fiber’s geometry, volume fraction, dispersion, arrangement, etc. Consequently, the modal expressions for the tensile strength or characteristic points in the tensile behavior generally consider such influential parameters [10,11,12,13,14,15,16,17,18,19]. The most common parameters used in these models are the fiber volume, fiber aspect ratio (length to diameter ratio), and fiber orientation. The first two parameters are determined in the process of material selection and mix design, whereas the last one is determined by considering the geometry of the structural member and how it should be placed during the placing process of fresh composites.

Even typical fiber-reinforced concrete (FRC) containing coarse aggregates exhibits some variations in fiber orientation according to the geometry of the structural member, and a fiber orientation coefficient is introduced to express its influence on the tensile strength and other properties. However, the impact of the fiber orientation on the tensile behavior of FRC is not as great as that of the fiber volume fraction or aspect ratio. When the fiber volume fraction is constant, the effect of fiber orientation distribution mainly due to manufacturing process is limited [17,18]. On the other hand, UHPCC does not have coarse aggregates and is composed of very fine particles (≤0.5 mm in diameter), which results in high fluidity and viscosity. This induces a certain preferential fiber orientation, much more than for typical FRC, resulting in a noticeable change in tensile strength and behavior. Therefore, in the case of UHPCC, the fiber orientation should be considered as a main parameter that has great influence on the mechanical properties [19,20,21,22].

Recommendations for the design and construction of UHPCC in Korea, Japan, France, etc. note that the effect of the fiber orientation should be considered when determining the characteristic tensile strength and behavior of the material for structural design [23,24,25]. According to the recommendations in France, the design strength is determined by dividing the characteristic strength by the fiber orientation coefficient, for which 1.25 for global effect and 1.75 for local effect may be applied to preliminary design when suitability tests are not conducted on a representative model of the actual structure, and it has to be substantiated at a later date [23]. Simon et al. [26] discussed the coefficient with extensive investigation for fiber distribution in several UHPCC projects and showed the robustness and reliability of the concept. They also reported that the coefficient was sometimes less than 1 or more than 2 in some cases.

The fiber orientation plays a major role in the mechanical properties, especially in the tensile behavior of UHPCC, as well as in the structural performance of UHPCC members as a result. In this regard, several researchers have examined the relationship between the tensile behavior and the fiber orientation distribution in UHPCC [19,20,21,27,28,29,30]. One of the authors also reported an analytical approach to predict the tensile behavior of UHPCC considering the fiber orientation distribution [21]. In order to evaluate the fiber orientation distribution or determine the coefficients related to it, several techniques have been applied. In the past, the number of fibers detected in a section was counted for this purpose and the fiber orientation distribution was estimated indirectly based on the relationship between the number of fibers and the fiber orientation distribution [19,31]. On the other hand, fiber orientation distribution is now being evaluated directly using image analysis techniques, with 2D or 3D images taken by a digital camera, microscope, or computed tomography (CT) [28,29,30,32,33,34,35,36,37]. Image analysis using 2D images is used most widely because the image can be taken easily and 2D images require relatively less complicated calculations for the analysis.

However, the accuracy of the fiber orientation distribution obtained from image analysis is influenced considerably by the image resolution and the influence becomes greater in the case of a more inclined fiber on the image [38,39,40]. Meanwhile, the number of fibers detected in an image is relatively accurate regardless of the image resolution, if an appropriate threshold value for detecting fiber is applied and the objects that might be misidentified as a fiber are removed.

In view of these points, this paper proposes an analytical method to predict the post-cracking tensile behavior of UHPCC with improved accuracy by considering both the fiber orientation distribution and the number of fibers detected simultaneously from the image analysis technique.

## 2. Experimental Program

### 2.1. Materials and Mix Design

UHPCC is composed of cement, sand, silica fume, filler, water, and some admixtures. The water/binder ratio used in this study was 0.2. A polycarboxylate-based superplasticizer was used to obtain the required workability for such a low water/binder ratio composition. Type I portland cement and undensified silica fume were used as the cementitious materials. Silica fume has the effect of increasing the strength via the pozzolanic reaction and filling the voids. Table 1 lists the physical and chemical properties of the cement and silica fume. Sand with a grain size below 0.5 mm and a density of 2.62 g/cm^3^ was used as the sole aggregate. In addition, the siliceous filler was used to improve the strength and workability. The filler had a mean grain size of approximately 4 μm, density of 2.62 g/cm^3^, and crystalline SiO_2_ > 98%. The particle size was in the intermediate range between that of cement and silica fume. Therefore, it produces an enhanced packing density of the UHPCC matrix, making it possible to obtain improved strength and workability. Table 2 lists the UHPCC mix proportion applied for this study. A total 2 vol % of straight steel fibers, whose tensile strength were 2500 MPa and density was 7.5 g/cm^3^, were incorporated. Two types of fibers with the same volume fraction were chosen; one fiber was 0.2 mm in diameter and 16.3 mm in length, and the other was 19.5 mm in length with the same diameter. In order to improve the tensile performance of the composites, the hybridization of two lengths of steel fibers was introduced in this study. As is well known, the long steel fiber can resist large cracks and the short steel fiber can resist small cracks.

### 2.2. Specimen Preparation and Experiment

**Figure 1:**
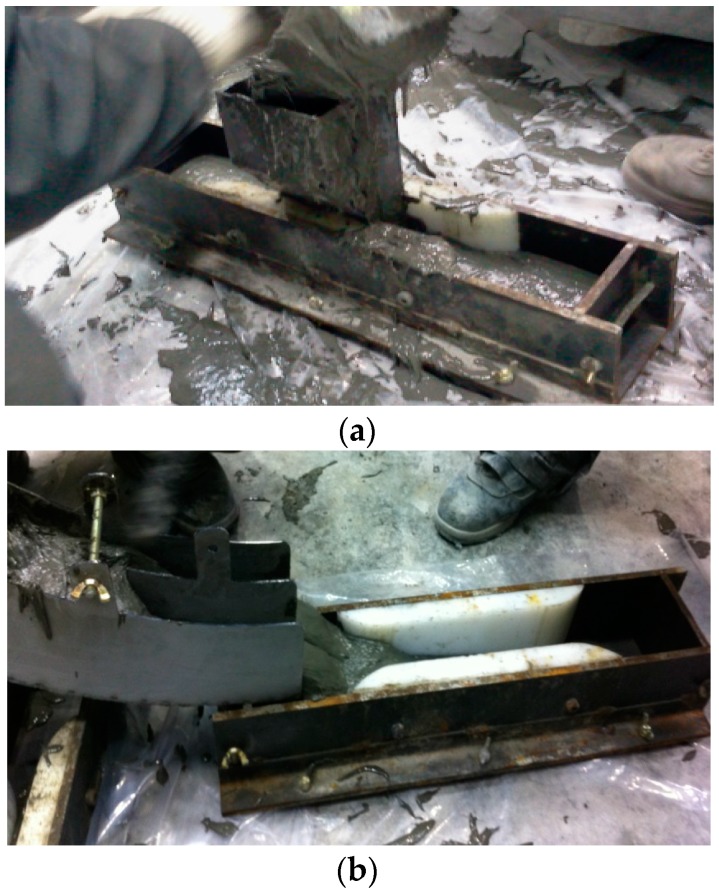
Two different casting methods: (**a**) Casting to induce a random fiber distribution in the middle of the specimen; (**b**) casting to induce fiber alignment along the flow direction.

In order to estimate the tensile behavior according to the fiber orientation distribution, two different fiber orientation distributions were induced by placing in different casting methods. One method was to cast in a way that could induce a random fiber distribution in the middle of the specimen, as shown in Figure 1a. The other was to cast in a way that could cause the fibers to align along the flow direction, as shown in Figure 1b. The former method is called ‘Method A’ and the latter is called ‘Method B’.

**Figure 2:**
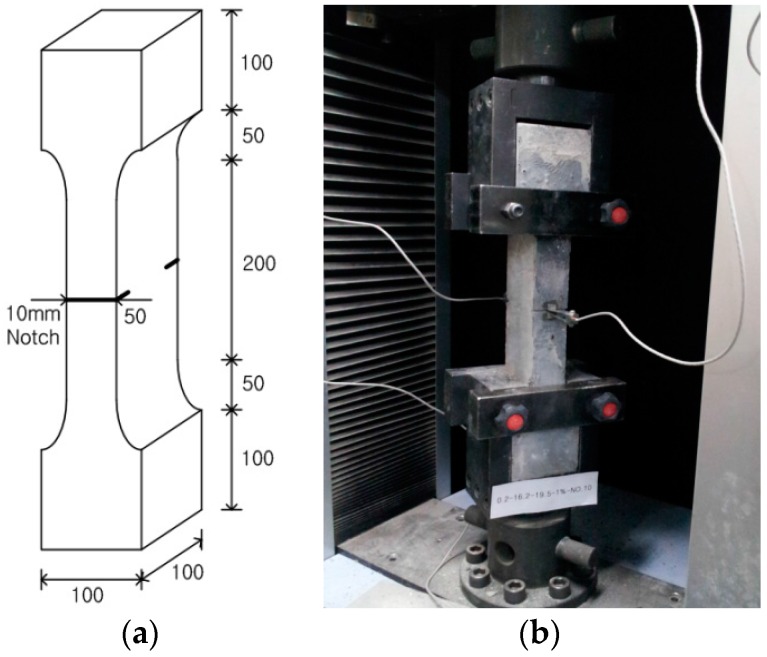
Specimen geometry and the setup for the direct tensile test: (**a**) The details of the specimen; (**b**) the experimental setup for the test.

For the direct tensile test experiment, the specimens were fabricated in the so-called dogbone shape. After casting, all the specimens were cured at room temperature for the first two days, demolded, cured in a steam curing regime at a temperature of 90 ± 3 °C for an additional three days, and stored in 20 ± 3 °C water until testing. Prior to the test, the specimens were notched by sawing on both sides at the center with a depth of 10 mm. Figure 2a provides the details of the notched specimen. The direct tensile test was implemented using a 250 kN capacity UTM with a loading rate of 0.2 mm/min. As each specimen was tested, its crack mouth opening displacement (CMOD) in the notch was measured using a clip gauge, which had been placed across the notch. The capacity of the clip gauge was 5 mm. Figure 2b shows the experimental setup for the test.

### 2.3. Test Results

**Figure 3:**
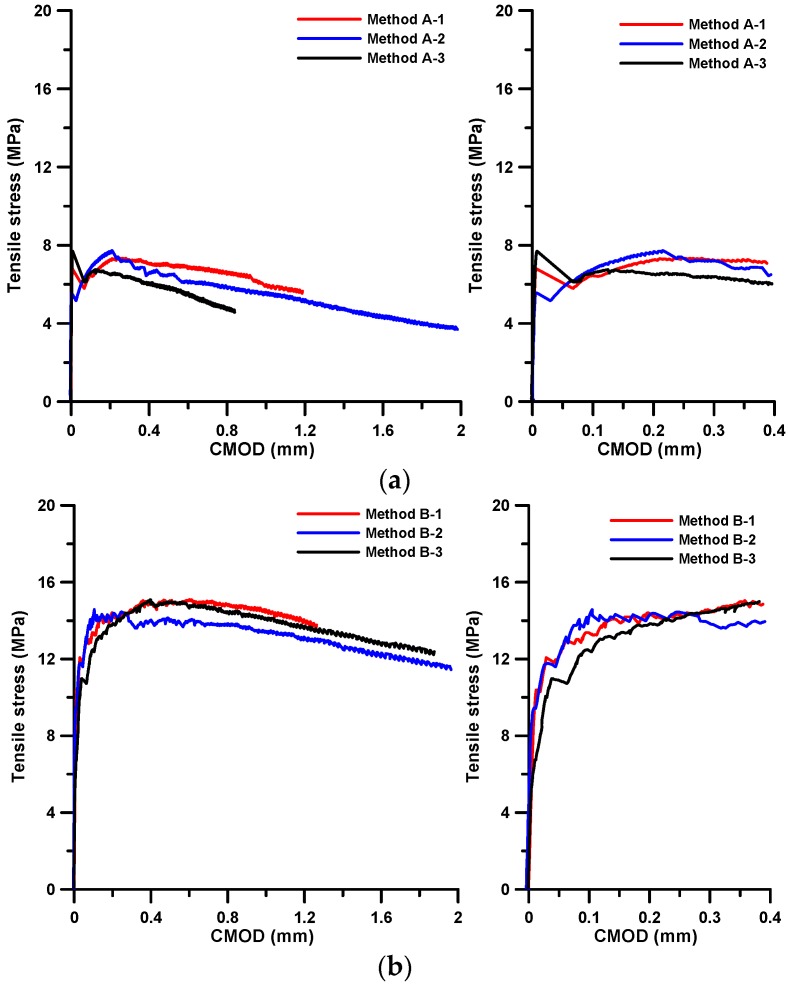
Tensile stress-CMOD curves for (**a**) Method A; and (**b**) Method B.

Figure 3 presents the tensile stress–CMOD curves obtained from the direct tensile test with the specimens cast in two different ways (i.e., Methods A and B). The figure clearly demonstrates that there is a noticeable difference in the tensile behavior according to the casting method.

The specimens, in which flow-induced fiber orientation was believed to be obtained, showed strain-hardening behavior until it reached a maximum tensile stress of approximately 15 MPa. In the other specimens, where the fiber orientation was not induced, there was a drastic decrease in stress once the first crack had occurred, and there was little stress recovery even though some of the stress appeared to have been regained for a little while after the first cracking. The maximum tensile stress did not reach 8 MPa. The first cracking stresses, tensile strengths (ultimate tensile stresses), and corresponding CMODs at those stresses or strength are tabulated in Table 3. The first cracking stress of Method B was slightly higher than that of Method A. It was theoretically proven that the distribution of fiber orientation has an influence on the first cracking stress even though its effect is quite limited and the strength of the matrix absolutely dominates the stress [18]. Meanwhile, the tensile strength of Method B was approximately twice that of Method A and the corresponding CMOD of Method B was also more than twice that of Method A. This difference in tensile behavior potentially indicates that the fiber orientation distribution depends strongly on the casting method and has a great influence on the post-cracking tensile behavior.

## 3. Determination of the Post-Cracking Tensile Behavior

### 3.1. Methodology

Once a crack forms in a fiber-reinforced cementitious composite, the matrix barely resists an external load at the cracked plane and most of the applied load is supported by the fibers. That is, its tensile behavior is determined absolutely by the pullout behaviors of the fibers embedded in the matrix.

**Figure 4:**
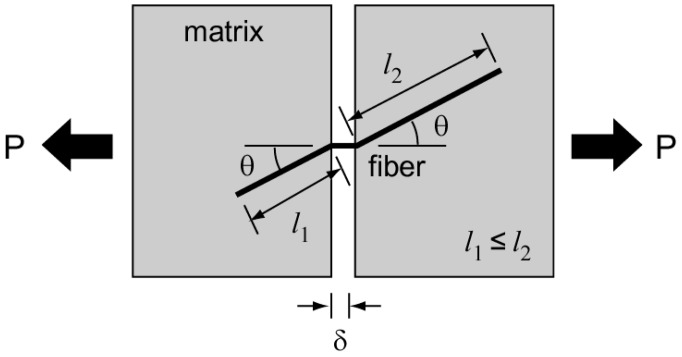
Embedded length of a fiber inclined with *θ* to the cracked plane.

A large number of fibers can be found across the cracked plane, and each fiber has its own embedded length and inclined angle from the cracked plane. Let us consider a single fiber embedded in a matrix across a crack plane with an inclined angle (*θ*), as depicted in Figure 4. For convenience, the embedded length (*l_e_*) is defined as the length of the shorter segment of the fiber when it is divided into two embedded segments due to cracking. It is therefore less than *l_f_*/2. It is well known that both the embedded length and inclined angle of the fiber have a strong influence on the pullout resistance, and the pullout force is also dependent on its pullout displacement or crack opening displacement on the cracked plane. Therefore, the bridging force of a single fiber shown in Figure 4 is given by a function of the inclined angle of the fiber (*θ*), the embedded length of the fiber (*l_e_*), and the crack opening displacement (*δ*), which is therefore denoted as *P*(*θ*, *l_e_*, *δ*). The fiber bridging stress of the composites after cracking, in which all the fibers placed with an embedded length and an inclination across the cracked plane are considered, can be calculated by the following equation [41]:

(1) $$\sigma_{b}\left(\delta \right)=\frac{4V_{f}}{\pi d_{f}^{2}}\int_{0}^{\frac{\pi }{2}}\int_{0}^{\frac{l_{f}}{2}}F_{c}\;P\left(\theta ,l_{e},\delta \right)\;p\left(l_{e}\right)\;p\left(\theta \right)\cos\theta \;dl_{e}\;d\theta ,$$

where *p*(*θ*) and *p*(*l_e_*) are the probability density functions for *θ* and *l_e_*, respectively. These two functions can be expressed by sin *θ* and 2/*l_f_* in particular for the three-dimensional random distribution of the fibers. *l_f_* and *d_f_* denote the length and diameter of the fiber, respectively.

Lee et al. [42] proposed a predictive model for the pullout behavior of an inclined steel fiber in a UHPCC matrix. The model they suggested in their work was applied for the pullout resistance of a single fiber. The bridging force of a single fiber, *P*(*θ*, *l_e_*, *δ*), can be obtained from the pullout resistance by assuming that (1) the crack width is equal to twice the slip deformation in the pullout behavior when the fiber is perfectly bonded and partially debonded; and (2) after the fiber is fully debonded, the crack width deformation is the same as the slip deformation because the shorter fiber only undergoes frictional slip deformation after debonding and is finally pulled out. More details of the function *P*(*θ*, *l_e_*, *δ*) can be found in [18]. In addition, in order to consider the inconsistency of the bond condition between that in their pullout test and in the composites, a correction factor (*F_c_*) was introduced and multiplied by the pullout resistance. A value of 1.25 was applied as this factor in an earlier study [18] and it can be changed according to the mix condition and the characteristics of the fiber used.

The post-cracking behavior of a composite can be defined as the combination of the resistance provided by the fibers and the matrix. Therefore, in order to establish the post-cracking behavior, a mathematical model for the resistance of the matrix should also be introduced in addition to fiber bridging. The typical tension softening behavior of concrete after cracking can be expressed by a linear, bilinear, or exponential curve. The tension softening curve of UHPCC in this study was assumed to be an exponential function [43]:

(2) $$\sigma_{ct}=f_{t}\exp\left(-cw\right),$$

where *f_t_* is the tensile strength (MPa), *w* is the cracking opening (mm), and *c* is a constant that can be determined experimentally and is theoretically equal to *f_t_*/*G_F_*.

### 3.2. Determination of Probability Density Distribution of Fiber Orientation

To derive the tensile bridging behavior of the fibers by Equation (1), considering the effect of the fiber orientation distribution, it is essential to determine the probability density function for the orientation of the fibers embedded in the composites. If the orientation distribution of the fibers embedded in the composites is measured directly, the measured distribution instead of the function can be applied for the term *p*(*θ*) in Equation (1).

**Figure 5:**
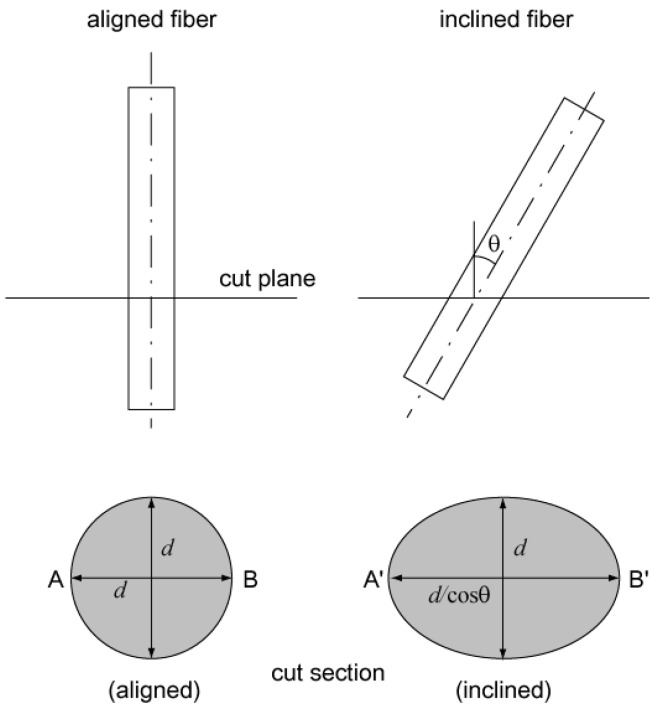
Schematic diagram for how to calculate fiber inclined angle.

After finishing the direct tensile tests, the tested specimens were sawn along a plane as near as possible to the fractured plane with a localized crack and the cut sections were ground; an image of the cut section for each specimen was then taken by a digital camera. The fiber orientation distribution was then obtained using the image analysis technique. The number of total pixels constituting a fiber section was at least 80 in the image, and the pixel size of the fiber diameter was in the range of 10~14 pixels. In the process of detecting fibers through the thresholding algorithm in the image analysis, 80 pixels for an object’s area were applied for the threshold to distinguish the fibers from other objects. The method for calculating the orientation of an inclined fiber is explained by Figure 5. Let us consider a fiber originally having a perfectly circular cross section. If it is laid at an inclined angle across a plane, the sectional geometry of the fiber on the plane becomes ellipsoidal and the ratio of the largest and smallest diameter in the ellipsoid is dependent on the inclination of the fiber. From Figure 5, the inclined angle (*θ*) of the fiber can be calculated using the following equation:

(3) $$\theta =\cos^{-1}\left(\frac{\bar{AB}}{\bar{A^{\prime }B^{\prime }}}\right).$$

**Figure 6:**
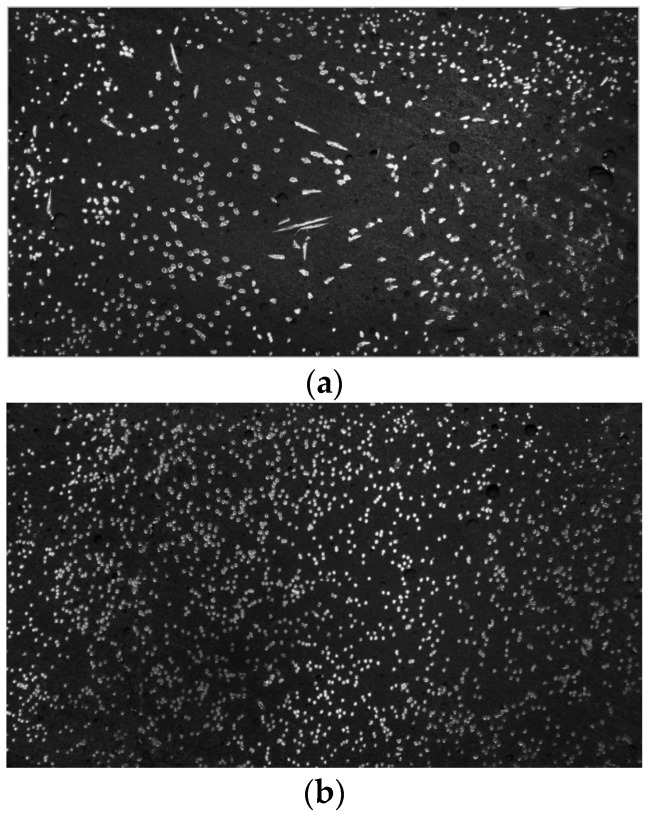
Images of the fiber distribution for the two different casting methods: (**a**) Method A; (**b**) Method B.

Figure 6 shows images of the sawn sections of the tested specimens. It can be easily seen that the number of fibers detected in the section image obtained from the specimen fabricated by Method B is much greater than in the Method A specimen. It can be also found that the fibers in the Method B section are mostly aligned in the direction normal to the plane, but Method A shows relatively fewer fibers as well as a higher percentage of fibers laid diagonally to the cut plane. This direct observation suggests that the differences in the fibers distributed in the cut section are closely related to the tensile behaviors of both cases.

**Figure 7:**
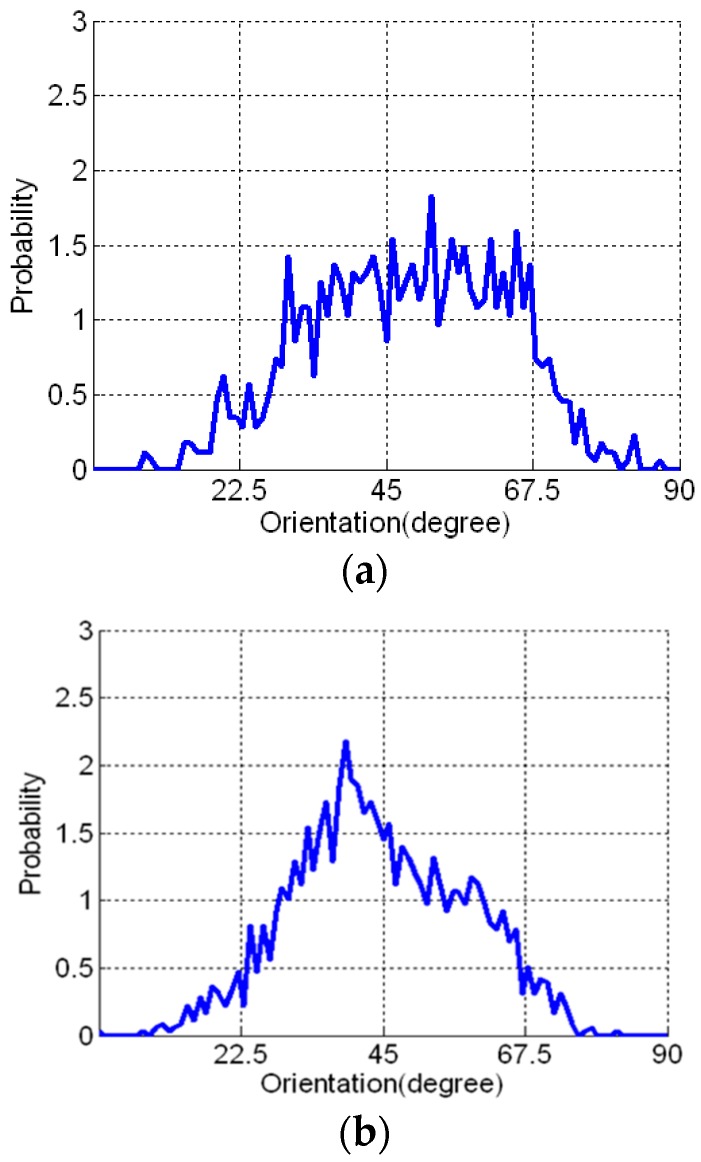
Fiber orientation distributions for the two different casting methods: (**a**) Method A; (**b**) Method B.

Table 4 presents the image analysis results. The average number of fibers calculated from the image analysis technique was 910 and 1872 for Methods A and B, respectively. The fiber orientation distribution for all detected fibers is given in Figure 7, in which the difference in the distributions is distinguishable but not as much as expected. In addition, higher intensity of distribution around 45° was shown, especially for the casting Method B. Two major reasons can be suggested for such fiber orientation distribution, as shown in Figure 7. First, according to hydrodynamics, most fibers subjected to shear flow are theoretically supposed to be aligned to the flow direction under the assumption that there is no interaction among fibers. The assumption may be applied for dilute suspensions of long rigid fibers and a composite with $V_{f}<1/r_{f}^{2}$ is classified into this category. $r_{f}$ means the aspect ratio, indicating the ratio of length to diameter of the fiber. Meanwhile, for semi-concentrated ($1/r_{f}^{2}\leq V_{f}\leq 1/r_{f}$) or concentrated ($V_{f}>1/r_{f}$) suspensions, the interaction among fibers cannot be neglected and the rotational movement of fibers is restricted in some degree [44]. The composites dealt with in this study have 2 vol % (fiber volume fraction) and therefore are classified into concentrated suspensions. The second is related to the error in calculating the orientation angle by image analysis, which is considerably dependent on the pixel size for expressing the fiber section. Lee et al. [40] investigated the error of the measured orientation angle of an artificial fiber image according to the number of pixels in the diameter and demonstrated that the error increased as the orientation angle and the number of pixels in the diameter of the fiber decreased. Table 5 presents the orientations calculated from fiber images with different orientation angle and the number of pixels in the study by Lee et al. [40]. It can be seen that the lower fiber orientation as well as the lower number of pixels caused the higher error in the measured orientation. In particular, the orientation in the range of 15°~45° was calculated as 41.2°~49.8° when the number of pixels in the diameter of the fiber was 5. Therefore, considering the pixel size of the fiber diameter was in the range of 10~14 pixels in this study, it can be said that the lower resolution of the fiber images might cause significant errors in calculated orientation distribution in the range of 0°~45°, which resulted in the intensive distribution around 45°.

From the measured distribution, it is possible to calculate the fiber orientation coefficient ($\eta_{\theta }$), which is defined as [45]

(4) $$\eta_{\theta }=\int_{0}^{\frac{\pi }{2}}\;p\left(\theta \right)\cos^{2}\theta \;d\theta .$$

The calculated mean fiber orientation coefficient was 0.447 for Method A and 0.531 for Method B.

Generally, the number of discontinuous short fibers detected in a cut plane is dependent on the fiber orientation coefficient [31]. A general equation for the number of fibers per unit area ($N_{f}$) is given by the following:

(5) $$N_{f}=\alpha_{f}\frac{V_{f}}{A_{f}},$$

where $V_{f}$ means the volume fraction of steel fibers in concrete, $A_{f}$ indicates the cross-sectional area of a steel fiber, and $\alpha_{f}$ presents an orientation factor accounting for its effect on the number of fibers. This value is in the range of 0.41 to 0.81 [31,46], and is equal to 1 when all the fibers are aligned in one dimension.

The orientation factor, $\alpha_{f}$, can be estimated indirectly from the calculated $N_{f}$ using Equation (5). The estimated $\alpha_{f}$ was 0.357 for Method A and 0.735 for Method B.

Li et al. [41] proposed an equation for the fiber number in a unit area bridging the cracked plane, which was expressed as follows:

(6) $$N_{f}=\frac{4V_{f}}{\pi d_{f}^{2}}\int_{0}^{\frac{\pi }{2}}\int_{0}^{\frac{l_{f}}{2}}\;p\left(l_{e}\right)\;p\left(\theta \right)\cos\theta \;dl_{e}\;d\theta .$$

By comparing Equations (5) and (6), the orientation factor, $\alpha_{f}$, can also be expressed as

(7) $$\alpha_{f}=\int_{0}^{\frac{\pi }{2}}\int_{0}^{\frac{l_{f}}{2}}\;p\left(l_{e}\right)\;p\left(\theta \right)\cos\theta \;dl_{e}\;d\theta .$$

With the measured orientation distribution, *p*(*θ*) for each case, the orientation factor can be calculated from Equation (7); the results are also listed in Table 4. The orientation factor for Method A is 0.645 and 0.715 for Method B. Compared to the values calculated with the detected fiber number and Equation (5), both approaches, using Equation (5) or (7), produced similar results to the orientation factors for Method B. On the other hand, Equation (7) for Method A led to a much lower value than Equation (5). This means that the number of detected fibers for Method A is much smaller than the one expected theoretically.

In the case that all the fibers are aligned in one direction, both $\eta_{\theta }$ and $\alpha_{f}$ are equal to 1. Considering the variation of the values for $\eta_{\theta }$ and $\alpha_{f}$ with two different casting methods, it can be said that $\alpha_{f}$ calculated from the number of detected fibers is a more sensitive indicator than $\eta_{\theta }$ and $\alpha_{f}$ obtained theoretically from the measured fiber orientation distribution, to the variation in the fiber orientation distribution.

### 3.3. Estimation of Post-Cracking Tensile Behavior

As mentioned earlier, the post-cracking tensile behavior can be defined by superposing the tension softening curve of the matrix and the bridging curve of the fibers.

The bridging resistance of the fibers was obtained from Equation (1) by applying the measured probability density function for the fiber orientation for the two different casting methods. Each bridging resistance developed by 1 vol % of 16.3 mm fibers and 1 vol % of 19.5 mm fibers was first obtained, and the total bridging resistance by the combination of two types of fibers was obtained by superposition. The function for the 16.3 mm and 19.5 mm fibers was assumed to be the same. The probability *p*(*l_e_*) in Equation (1) was assumed to be 2/*l_f_*. It may not strictly follow the uniform distribution from 0 to *l_f_*/2, but to our knowledge there is no feasible method to determine *p*(*l_e_*). In the calculation, the model for the pullout resistance suggested by Lee et al. [42] was modified slightly to fit the experimental data. The values employed in this study for the parameters in the model were listed in Table 6. Two parameters (*γ* and *n*) describing the slip coefficient for the ascending branch of the pullout behavior were only adjusted to fit the experimental data. According to the analysis based on the model, the slip deformation at the peak pullout load for a fiber with an inclined angle is larger for longer fibers considering the difference in the bond stress distribution along the interface between fiber and matrix at the peak load. The correction factor (*F_c_*) in Equation (1) introduced for considering the variation due to the difference in the bond condition had a value of 1.2 in the analysis.

Once each bridging stress–crack width curve of the two types of fibers laid across the cracked plane is obtained, the tensile bridging behavior developed by incorporating the two kinds of fibers can be plotted by superposing each bridging curve for both fibers.

**Figure 8:**
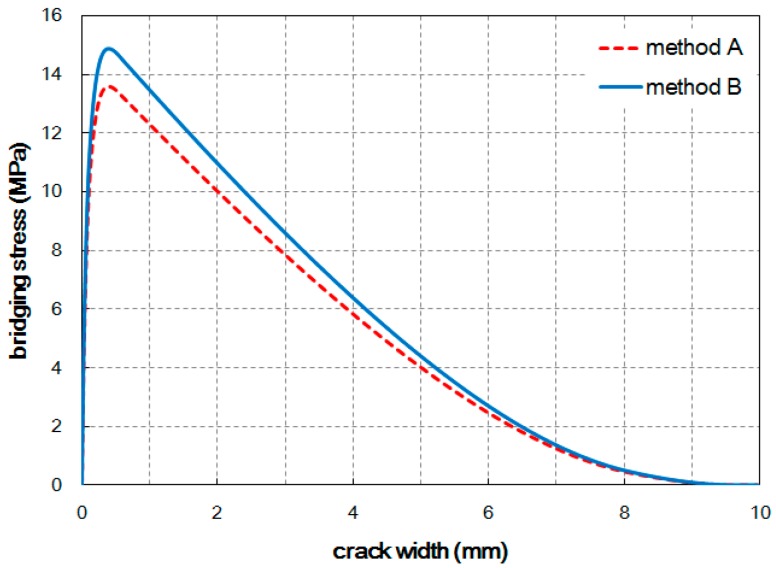
Tensile bridging behaviors estimated from the image analysis results.

The tensile bridging behavior for a representative specimen of each case was obtained. The specimen that had a median value and the value closest to the average for most of the parameters listed in Table 3 was selected as representative for each case. Figure 8 shows the estimated tensile bridging behaviors. The maximum bridging stress for Method A and Method B was 13.6 MPa and 14.9 MPa, respectively. Some difference in the tensile bridging behaviors, due to the difference in the fiber orientation distribution, can be seen. However, compared with the tensile strengths obtained from experiment, the difference is much smaller. This means that the fiber orientation distribution measured from the image analysis does not completely represent the influence of the placing method, and the fiber distribution and consequent tensile behavior vary according to the placing method.

In addition, the calculated maximum bridging stress for Method A was overestimated considerably. This seems to be closely related to the number of detected fibers. The theoretically estimated number of fibers per unit area (0.715 ×$\;4V_{f}/\pi d_{f}^{2}$) was similar to the measured one (0.735 ×$\;4V_{f}/\pi d_{f}^{2}$) for the case of Method B, whereas Method A resulted in a huge difference between the theoretically estimated number of fibers per unit area (0.644 ×$\;4V_{f}/\pi d_{f}^{2}$) and the measured one (0.357 ×$\;4V_{f}/\pi d_{f}^{2}$). This is believed to have been caused by several factors, including the inaccuracy in calculating the inclined angle of the highly inclined fibers, due to the limited number of pixels in the image used in the image analysis technique, and the consequent inaccuracy in the fiber orientation distribution, *p*(*θ*).

**Figure 9:**
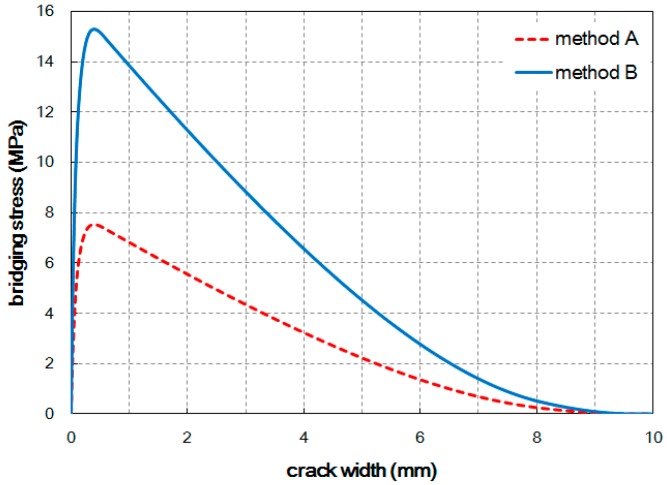
Modified tensile bridging behaviors after considering the number of detected fibers.

The number of fibers detected in an image is relatively accurate regardless of the image resolution, as explained previously. If the difference in the number of fibers is therefore considered when estimating the tensile bridging behavior, more reasonable results can be obtained. By multiplying Equation (1) by the ratio of the orientation factor ($\alpha_{f}$) obtained from Equations (5) and (7), the tensile bridging behaviors can be modified; the results are plotted in Figure 9. After modification, the maximum bridging stresses became similar to the measured tensile strengths for both cases. Therefore, the number of fibers placed across the cracked plane provides better tensile responses, similar to the measured tensile stress–CMOD curves.

The tension softening curve of the matrix needs to be defined to calculate the post-cracking tensile behavior of UHPCC. Kang and Kim [21] introduced the concept of apparent tension softening curve and fracture energy in order to model the post-cracking tensile behavior of UHPCC by superposing the resistance of the bridging fibers and the tension softening of the matrix. In their research, it was shown that the post-cracking tensile resistance of cementitious matrix in a fiber-reinforced cementitious composite was much higher than its own tension softening curve. Therefore, when a much higher fracture energy than the intrinsic value for the tension softening curve was applied, the estimated behaviors became more consistent with the experimental results. It was also said that this apparent tension softening curve might be dependent on the fiber characteristics as well as the matrix properties. For the apparent tension softening curve of the matrix in this study, the tensile strength, *f_t_* was assumed to be 6.68 for Method A and 7.55 for Method B, which were determined from the experimental results. The fracture energy, *G_F_*, for determining *c* was assumed to be 0.5 N/mm, which was determined under the consideration of the magnitude of the stress drop after first cracking and the corresponding crack width in the tensile behavior of UHPCC.

**Figure 10:**
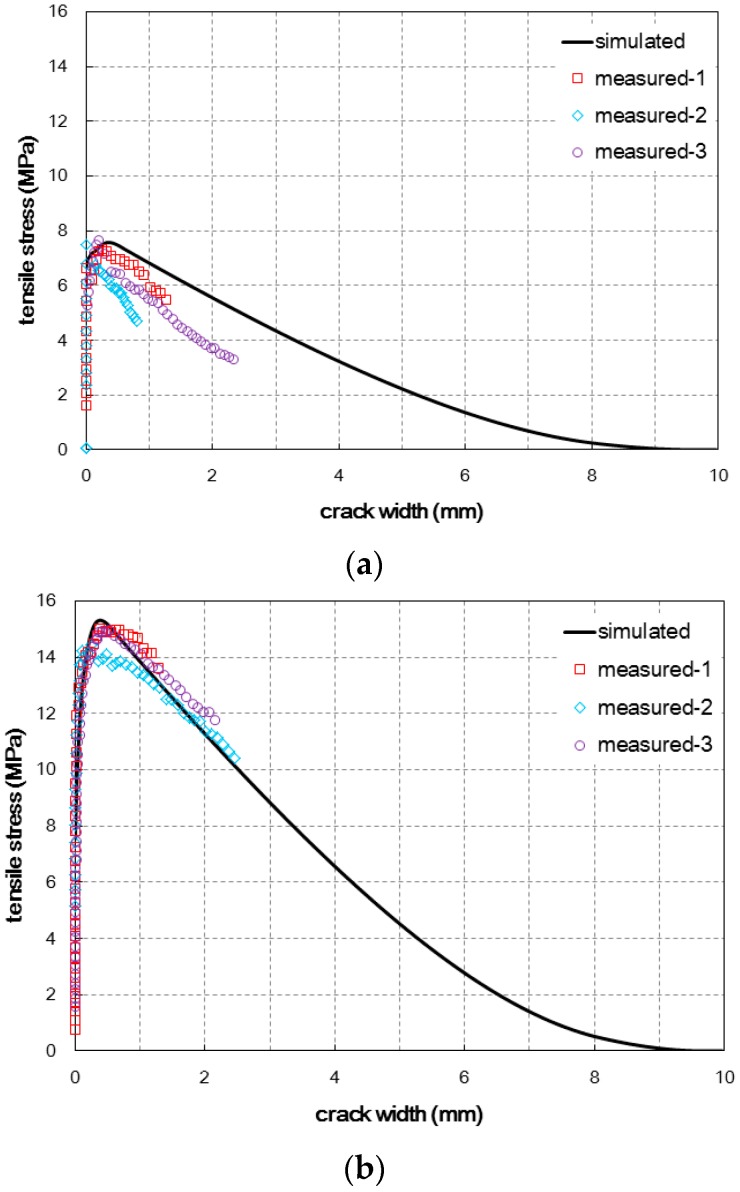
Comparison of the simulated and measured tensile behaviors for (**a**) Method A and (**b**) Method B.

When the estimated tensile behaviors were compared with the experimental results, as shown in Figure 10, the estimation results provided acceptable agreement with the measured tensile behaviors for both specimens fabricated with the two different casting methods. The slope in the softening branch, tensile hardening behavior after the first cracking, and tensile strength could be estimated properly. With regard to the crack width at the ultimate tensile stress, the simulated crack width was similar for the two cases but the experimental results presented a noticeable difference between the two cases. The crack width for Method B was larger than the value for Method A. This inconsistency may be ascribed to the inaccuracy of the assumed fiber pullout behavior according to the inclined angle. Especially for Method A, the overestimation of the pullout deformation of highly inclined fibers is thought to cause the difference between the simulated and measured values. If a more accurate model for the fiber pullout behavior according to the inclined angle is provided based on experiments, the prediction of the crack width at the ultimate tensile stress may also be improved.

Through the analysis and comparison with the experimental results for the post-cracking tensile behavior, the validity of the approach adopted in this study to simulate the post-cracking behavior of fiber-reinforced cementitious composites was proven.

## 4. Conclusions

In this study, the post-cracking tensile behavior of UHPCC according to the fiber orientation distribution was examined and an improved analytical model for better prediction of the behavior depending on the fiber orientation distribution was suggested.

In order to induce two different fiber orientation distributions and consequently different uniaxial tensile behaviors of UHPCC, two different casting methods were adopted. One method (named ‘Method A’) was to achieve a random fiber distribution in the middle of the specimen, and the other (named Method B’) was to induce a fiber arrangement that was closely aligned along the tensile force direction. The direct tensile test results showed that the post-cracking tensile behavior was considerably dependent on the casting method. The tensile strength of Method B was approximately twice that of Method A. The influence of each casting method was quantified by image analysis for the fiber distribution. Differences in the number of fibers detected, fiber orientation distribution, and relevant parameters could be demonstrated. The number of fibers detected in the section image of Method B was much greater than for Method A, while the difference in the fiber orientation distributions was still distinguishable but not as much as expected.

From the obtained fiber orientation distribution, the post-cracking tensile behavior of the fiber-reinforced cementitious composites was simulated. When only the fiber orientation distribution was considered in the simulation, the predicted behaviors differed from the behaviors measured from the experiment, particularly in the case of Method A. This means that the fiber orientation distribution measured from the image analysis does not completely represent the influence of the placing method, and the fiber distribution and consequent tensile behavior varied according to the placing method.

A comparison of the number of fibers counted from the sectional image and the number calculated theoretically based on the fiber orientation distribution measured from image analysis presented a remarkable difference in the case of Method A, which was as much as the difference in the simulated and measured tensile strengths of Method A. Considering the number of fibers detected in an image is relatively accurate regardless of the image resolution, this indicates that the limited resolution of the fiber images might cause significant errors in calculated orientation distribution. When the discrepancy in the number of fibers detected and expected from the measured fiber orientation distribution was also considered in addition to the fiber orientation distribution, more reasonable behaviors similar to the measured ones could be obtained for both casting methods. The slope in the softening branch, tensile hardening behavior after the first cracking, and tensile strength could all be estimated properly.

## Figures and Tables

**Table 1:** Physical and chemical properties of cement and silica fume.

Item	Specific Surface Area (cm^2^/g)	Density (g/cm^3^)	Ig.loss (%)	Chemical Composition (%)					
				SiO_2_	Al_2_O_3_	Fe_2_O_3_	CaO	MgO	SO_3_
Cement	3413	3.15	1.40	21.01	6.40	3.12	61.33	3.02	2.3
Silica fume	200,000	2.10	1.50	96.00	0.25	0.12	0.38	0.1	-

**Table 2:** Mix proportion of UHPCC.

Unit Mass (kg/m^3^)						
Cement	Silica Fume	Sand	Filler	WRA *	Water	Steel Fiber
771	193	848	231	46.3	160	156

* WRA: Water reducing agent.

**Table 3:** Results of the direct tensile test.

Specimen	At First Cracking		At Ultimate Stress	
	Stress (MPa)	CMOD (mm)	Stress (MPa)	CMOD (mm)
Method A-1	6.79	0.007	7.34	0.249
Method A-2	5.56	0.007	7.73	0.215
Method A-3	7.70	0.008	7.70	0.008
Average	6.68	0.007	7.59	0.157
St. dev.	1.07	0.001	0.217	0.130
Method B-1	8.69	0.008	15.10	0.601
Method B-2	8.08	0.008	14.58	0.109
Method B-3	5.88	0.006	15.09	0.397
Average	7.55	0.007	14.92	0.369
St. dev.	1.48	0.001	0.297	0.247

**Table 4:** Image analysis results for the fiber distribution.

Specimen		The Number of Total Fibers Detected	The Number of Fibers per Unit Area (*N_f_*/mm^2^)	$\eta_{\theta }$ (Equation (4))	$\alpha_{f}$	
					Equation (5)	Equation (7)
Method A	1	821	0.205	0.418	0.322	0.620
	2	988	0.247	0.475	0.388	0.667
	3	920	0.230	0.448	0.361	0.646
	Mean	910	0.227	0.447	0.357	0.645
Method B	1	1842	0.406	0.501	0.723	0.692
	2	1927	0.481	0.521	0.757	0.707
	3	1847	0.461	0.572	0.725	0.745
	Mean	1872	0.468	0.531	0.735	0.715

**Table 5:** Measured orientation angle of the artificial fiber image according to the number of pixels in the diameter [40].

Number of Pixels in the Diameter of the Fiber	Fiber Orientation Angle (°)				
	0	15	30	45	60
5	22.2	41.2	43.2	49.8	62.3
25	6.6	18.7	30.2	45.7	59.9
50	4.3	14.5	29.6	44.4	59.6
100	1.7	14.7	29.9	44.9	59.9

**Table 6:** The values employed in this study for the parameters in the pullout model by Lee et al. [42].

Component		Parameter	Value	Description
Material properties	Matrix	$E_{m}$	45	Elastic modulus (GPa)
		$\upsilon_{m}$	0.2	Poisson’s ratio
	Fiber	$E_{f}$	200	Elastic modulus (GPa)
		$\upsilon_{f}$	0.3	Poisson’s ratio
		$\tau_{\max\left(app\right)}$	6.8	Apparent maximum bond strength (MPa)
		$\tau_{f\left(app\right)}$	6.8	Apparent frictional bond strength (MPa)
For ascending branch of the pullout behavior		f	1.6	Snubbing friction coefficient
		κ	1.8	Spalling coefficient
		γ	5	Parameters describing slip coefficient
		n	0.4	
For descending branch of the pullout behavior		η	0.05	Parameters related to the shape of the branch
		α	1.0	
