# Erratum: Myoung, S.C.; Su-Tae, K.; Bang, Y.L.; Kyeong-Taek, K.; Gum-Sung, R. Improvement in Predicting the Post-Cracking Tensile Behavior of Ultra-High Performance Cementitious Composites Based on Fiber Orientation Distribution. *Materials* 2016, *9*, 829

**DOI:** 10.3390/ma10010063

**Published:** 2017-01-19

**Authors:** Myoung Sung Choi, Su-Tae Kang, Bang Yeon Lee, Kyeong-Taek Koh, Gum-Sung Ryu

**Affiliations:** 1 Department of Safety Engineering, Dongguk University-Gyeongju, 123 Dongdae-ro, Gyeongju, Gyeongbuk 38066, Korea; mschoi@dongguk.ac.kr; 2 Department of Civil Engineering, Daegu University, 201 Daegudae-ro, Jillyang, Gyeongsan, Gyeongbuk 38453, Korea; 3 School of Architecture, Chonnam National University, 77 Yongbong-ro, Buk-gu, Gwangju 61186, Korea; bylee@jnu.ac.kr; 4 Structural Engineering Research Institute , Korea Institute of Civil Engineering and Building Technology, 283 Goyangdae-Ro, Ilsanseo-Gu, Goyang, Gyeonggi 10223, Korea; ktgo@kict.re.kr (K.-T.K.); ryu0505@kict.re.kr (G.-S.R.)

1. The authors wish to remove the symbol “†”, which indicates the equal contribution from each author. The correct authorship is shown below:

Myoung Sung Choi ^1^, Su-Tae Kang ^2,^*, Bang Yeon Lee ^3^, Kyeong-Taek Koh ^4^ and Gum-Sung Ryu ^4^

2. The authors also wish to update the “Author Contributions” part, the correct one is shown below:

**Author Contributions:** Myoung Sung Choi, he did all of the experiments, did the analysis of the data and wrote the manuscript. Su-Tae Kang was the principle investigators and supervised the whole work. Bang Yeon Lee assisted the experiments and helped to analysis the data. Kyeong-Taek Koh helped to analysis the data and wrote the some parts of Chapter 3. And Gum-Sung Ryu assisted the experiments and wrote the some parts of Chapter 3.

The authors are responsible for these errors, they regret any inconvenience or misunderstanding caused by them.
